# Efficient Removal of Pb(Ⅱ) by Highly Porous Polymeric Sponges Self-Assembled from a Poly(Amic Acid)

**DOI:** 10.3390/molecules28072897

**Published:** 2023-03-23

**Authors:** Ying Leng, Kai Jin, Tian Wang, Xiaoyong Lai, Hui Sun

**Affiliations:** 1 State Key Laboratory of High-Efficiency Coal Utilization and Green Chemical Engineering, School of Chemistry and Chemical Engineering, Ningxia University, Yinchuan 750021, China; 2 Department of Chemistry, University of Washington, Seattle, WA 98195, USA

**Keywords:** water remediation, Pb(II) adsorption, polymeric sponge, self-assembly, poly(amic acid)

## Abstract

Lead (II) (Pb(II)) is widespread in water and very harmful to creatures, and the efficient removal of it is still challenging. Therefore, we prepared a novel sponge-like polymer-based absorbent (poly(amic acid), PAA sponge) with a highly porous structure using a straightforward polymer self-assembly strategy for the efficient removal of Pb(II). In this study, the effects of the pH, dosage, adsorption time and concentration of Pb(II) on the adsorption behavior of the PAA sponge are investigated, revealing a rapid adsorption process with a removal efficiency up to 89.0% in 2 min. Based on the adsorption thermodynamics, the adsorption capacity increases with the concentration of Pb(II), reaching a maximum adsorption capacity of 609.7 mg g^−1^ according to the Langmuir simulation fitting. Furthermore, the PAA sponge can be efficiently recycled and the removal efficiency of Pb(II) is still as high as 93% after five adsorption–desorption cycles. Fourier transform infrared spectroscopy and X-ray photoelectron spectroscopy analyses reveal that the efficient adsorption of Pb(II) by the PAA sponge is mainly due to the strong interaction between nitrogen-containing functional groups and Pb(II), and the coordination of oxygen atoms is also involved. Overall, we propose a polymer self-assembly strategy to easily prepare a PAA sponge for the efficient removal of Pb(II) from water.

## 1. Introduction

Clean water is very important for the survival of plants and animals. However, with the rapid development of industrial and social modernization, the shortage of clean water and water pollution caused by various contaminants including dyes [1], polycyclic aromatic hydrocarbons (PAHs) [2] and heavy metal ions [3], etc., have brought severe global crises [4]. Among the many pollutants, heavy metal ions have drawn particular interest to scientific fields such as chemistry, nanotechnology and materials due to their features of widespread, persistent and high toxicity [5]. Lead (II) ion (Pb(II)) is considered one of the most harmful pollutants even at trace level concentration because of its widespread presence in the environment, accumulation and high toxicity to children [6]. If ingested in excess, lead can cause damage to the central nervous system, kidney, gastrointestinal, hematopoietic, cardiovascular and reproductive systems, and to brain function [7]. Therefore, the efficient removal of Pb(II) from water is of high urgency and great significance.

Various methods, including chemical precipitation, physical adsorption, ion exchange, membrane filtration, and so forth, are widely used to treat Pb(II) contaminated water [5,8,9,10,11,12,13], among which physical adsorption is one of the most efficient methods [14]. In comparison with the above-mentioned methods, physical adsorption is usually easy to carry out, cheap, recyclable and effective [5]. In particular, the rapid development of nanotechnology brings the opportunity for the booming of nano-adsorbents, which have emerged as one of the most promising candidates to remove Pb(II) from water [15]. A large amount of functional nanomaterials, including carbon-based nanomaterials, chitosan [16], metal–organic frameworks (MOFs) [17,18,19,20], micelles [21,22], vesicles [23], magnetic nanoparticles [24,25,26,27], silica nanoparticles [28,29] and so forth, have been well developed to capture Pb(II) and other heavy metal ions from water. Recently, polymer self-assembly has been rapidly developed as a powerful tool to prepare functional nanomaterials [4,30,31,32]. For instance, our group presented a multifunctional homopolymer vesicle self-assembled from a scalable homopolymer, poly(amic acid) (PAA), to effectively remove four kinds of heavy metal ion (Ni^2+^, Cu^2+^, Zn^2+^, Pb^2+^) from wastewater [33]. However, it has always been hard to remove the nano-adsorbents from water after adsorption, which hinders the wider application of these functional nano-adsorbents. 

**Scheme 1:**
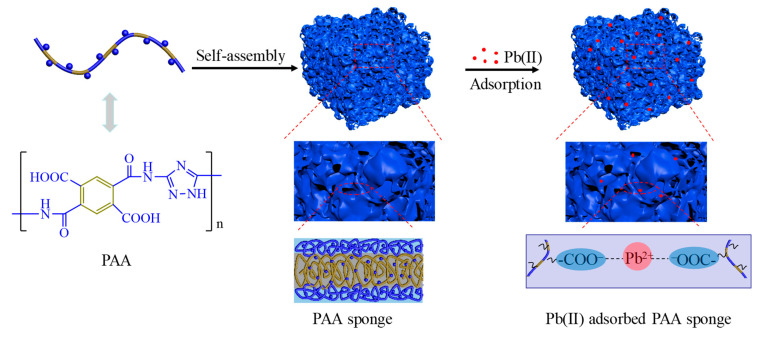
Schematic illustration of the preparation of the PAA sponge for the efficient adsorption of Pb(II). The blue dots represent the charged carboxylic group. The blue line represents the hydrophilic parts of PAA, while the yellow line represents the hydrophobic parts of PAA.

Herein, we prepare a novel highly porous sponge-like nanostructure (PAA sponge) derived from the self-assembly of a poly(amic acid) (PAA) for the efficient removal of Pb(II) from water. As shown in Scheme 1, the amphiphilic alternating copolymer PAA was prepared by the condensation polymerization of two commercially available monomers, pyromellitic dianhydride (PMDA) and 3,5-diamino-1,2,4-triazole, under mild condition, which could self-assemble into nano-sponges with hydrophobic aromatic groups (yellow color) forming the framework and the hydrophilic moieties (blue color) covering the surface. Taking advantage of the high porosity and ultrathin frameworks of PAA sponge and functional groups that have high affinity with Pb(II), such as carboxylic and triazole groups, the PAA sponge shows excellent performance when removing Pb(II) from water via electrostatic interaction, coordination and chelation. Notably, more than 89.0% of Pb(II) can be removed from water within 2 min with a maximum adsorption capacity of 609.7 mg g^−1^. In addition, FTIR and XPS were also used to confirm the strong interaction between Pb(II) and the functional groups. 

## 2. Results and Discussion

### 2.1. Synthesis and Self-Assembly of PAA

**Figure 1:**
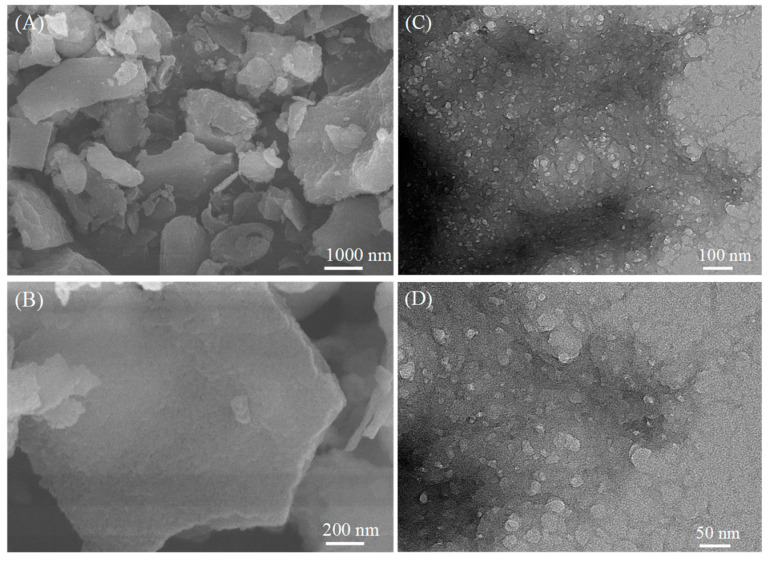
Morphology characterizations of PAA sponge: (**A**,**B**) SEM and (**C**,**D**) TEM images.

The synthesis of PAA was carried out according to our previous publication, as shown in Scheme S1 [34,35]. Fourier transform infrared spectroscopy (FTIR) and proton nuclear magnetic resonance (^1^H NMR) were conducted to confirm the structure of PAA (Figures S1 and S2) [34,35]. The PAA sponge was prepared by directly adding deionized water to the PAA solution (30 mg mL^−1^), diluted by the stock solution with *N*,*N*-dimethylformamide (DMF). The organic solvent was removed by dialysis against deionized water, and the morphology of the PAA sponge was observed using a scanning electron microscope (SEM) and a transmission electron microscope (TEM), demonstrating a sponge-like structure with high porosity and an ultrathin skeleton, as illustrated in Figure 1A–D. The formation process and the structure of the PAA sponge was proposed as follows: with the addition of deionized water to the DMF solution of PAA, the phase separation between the hydrophilic and hydrophobic parts of PAA drove the formation of very thin polymer membranes, as presented in Figure S3. Then, the thin membrane self-assembled into the PAA sponge with hydrophobic segments formed the skeleton and hydrophilic components covering the surface of the skeleton to maintain the colloidal stability by electrostatic repulsion (Scheme 1). The zeta potential of the PAA sponge was measured to be −28.2 mV, demonstrating the strong negatively charged surface (Figure S4). The specific surface area of the PAA sponge was calculated to be 23 m^2^ g^−1^ (Figure S5A), and the existence of micro- and mesopores was also confirmed by TEM images (Figure 1C,D) and pore distributions in Figure S5B. The formation of the PAA sponge by the self-assembly of PAA with a highly porous structure and charged hydrophilic surface would be beneficial for the diffusion, sufficient contact and adsorption of Pb(Ⅱ).

### 2.2. Effect of pH and PAA Sponge Dosage on Adsorption Performance

**Figure 2:**
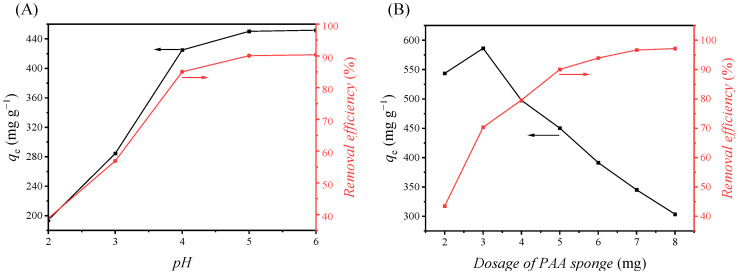
Effect of (**A**) pH and (**B**) dosage on the adsorption capacity at equilibrium (*q*_e_) and removal efficiency of the PAA sponge toward Pb(II).

The pH of the solution is a key parameter that determines the adsorption performance toward heavy metal ions because of the surface charges and functional group chemistry of the adsorbent, which also has critical influence on the zeta potential and colloidal stability of the nanomaterials. As illustrated in Figure S6, when the solution is strongly acidic (pH 2), the zeta potential increased from −28.2 to −17.8 mV, implying the reduction of negative charges on the surface due to the protonation of carboxylic groups. With the increase of pH, part of carboxylic groups deprotonated, leading to the enhancement of negative charges on the surface of the PAA sponge, showing the decrease in zeta potentials (Figure S6). When the pH was 5, the zeta potential reached the minimum of −33.6 mV, which would be beneficial for the adsorption of Pb(II) due to strong electrostatic interaction. Indeed, the adsorption capacity and removal efficiency also exhibited a similar trend, as shown in Figure 2A. When the pH of the solution was 2, the adsorption capacity and removal efficiency were only 194.2 mg g^−1^ and 38.8%, respectively. With the increase of pH, the adsorption capacity and removal efficiency continuously increased to 450.3 mg g^−1^ and 90.0% at pH 5, respectively. As the pH further increased to 6, the adsorption capacity and removal efficiency barely changed, demonstrating the saturation of adsorption due to the sufficient deprotonation of carboxylic groups. In addition, we also confirmed that the adjustment of the pH would not lead to the precipitation of Pb(II). The concentrations of lead(II) were 99.6 ppm and 100.3 ppm at pH 2 and 5, respectively, as measured by ICP-OES. Therefore, we conducted the following adsorption experiments at pH 5. 

The dosage of the PAA sponge also has an important impact on the adsorption behavior of Pb(II). As shown in Figure 2B, the adsorption capacity decreased with the dosage of the PAA sponge, while the removal efficiency showed the opposite trend. When the dosage of the PAA sponge was low (≤4.0 mg), even if the adsorption capacity was pretty high (above 500 mg g^−1^) the removal efficiency was lower than 80%, demonstrating a residual concentration of Pb(II) still as high as 20.4 ppm. As the dosage of the PAA sponge increased to 6 mg, the removal capacity decreased to 391.5 mg g^−1^, while the removal efficiency increased to 94.0%. With the further increase of the dosage to 8 mg, the removal efficiency reached 97.2% with an adsorption capacity of 303.6 mg g^−1^, which is much better than previous studies [36,37,38,39], demonstrating the excellent adsorption performance of the PAA sponge towards Pb(II). It was noteworthy that the PAA sponge could be precipitated from the solution after adsorption due to the neutralization of the surface negative charges, as shown in Figure S7, which avoided the tedious removal procedures of nano-adsorbents.

### 2.3. Adsorption Kinetics

To further investigate the adsorption kinetics of Pb(II) ions by PAA sponge, the adsorption kinetics of Pb(II) were fitted by pseudo-first-order and pseudo-second-order kinetic models, respectively. The pseudo-first-order model described the initial stage of the adsorption process when the initial concentration was high, and the adsorbents had a few active sites. In that case, the external diffusion or the internal diffusion are the rate controlling step. The pseudo-second-order model reflects the final stage of the adsorption when the initial concentration is low. There are abundant active sites on the adsorbents, in which the adsorption kinetics are dominated by the adsorption onto the active site [40].

The pseudo-first-order kinetic model:

(1) $$ln(q_{e}-q_{t})=lnq_{e}-k_{1}t$$

The pseudo-second-order kinetic model:

(2) $$\frac{t}{q_{t}}=\frac{1}{{q_{e}^{2}k}_{2}}+\frac{t}{q_{e}}$$

where *q*_t_ (mg g^−1^) and *q*_e_ (mg g^−1^) are the adsorption capacity of Pb(II) at time *t* (min) and equilibrium, respectively; *k*_1_ (min^−1^) and *k*_2_ (g mg^−1^ min^−1^) show the reaction rate constant of the pseudo-first-order and the pseudo-second-order kinetic models, respectively.

**Figure 3:**
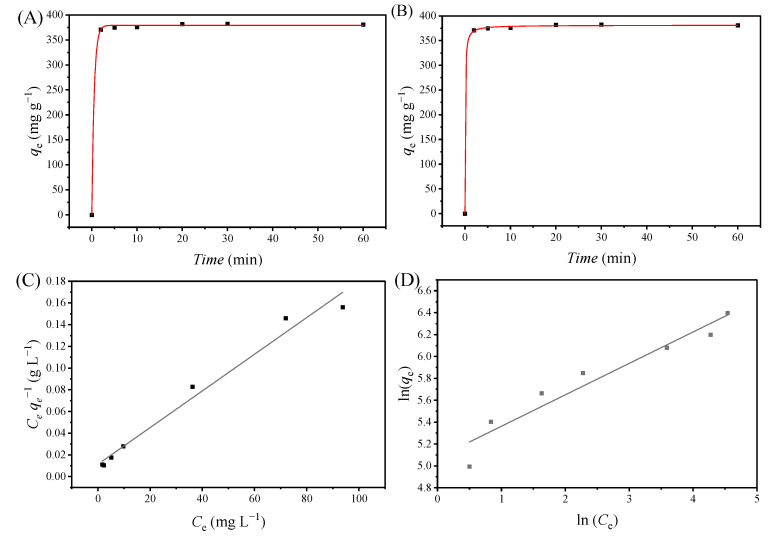
The adsorption kinetics of Pb(II) by PAA sponge fitted by (**A**) pseudo-first-order and (**B**) pseudo-second-order models, and the linear regression of the adsorption isotherm by fitting the equilibrium data with (**C**) the Langmuir and (**D**) the Freundlich adsorption models. The kinetic parameters and the thermodynamic parameters are presented in Table 1 and Table 2.

At the initial concentration of Pb(II) of 100 ppm and pH 5, the adsorption kinetics of Pb(II) by PAA sponge at different adsorption times were investigated. As shown in Figure S8, the adsorption process of Pb(II) was very fast. Additionally, the removal efficiency reached 89.0% with an adsorption capacity of 370.7 mg g^−1^ within 2 min, which we believe could be ascribed to the excellent water dispersability, highly porous structure and abundant functional groups on the surface of the PAA sponge, facilitating the fast adsorption of Pb(II). The adsorption reached equilibrium after 20 min with a removal efficiency of 91.7%. In order to investigate the adsorption kinetics quantitatively, we fitted the adsorption curve with pseudo-first-order and pseudo-second-order kinetic models, as illustrated in Figure 3A,B. Both the pseudo-first-order and pseudo-second-order kinetic models could be fitted well with the adsorption curve, showing excellent correlation. The fitting parameters are summarized in Table 1, and the correlation coefficient (*R*^2^) reached 0.9995 and 0.9998 for pseudo-first-order and pseudo-second-order kinetic models, respectively. In addition, the calculated *q*_e_ (378.3 and 381.4 mg g^−1^) was also well fitted with the experimental result (378.4–382.4 mg g^−1^). Therefore, we can conclude that the adsorption of Pb(II) is dominated by both of physisorption and chemisorption [41].

**Table 1:** Adsorption kinetics parameters of the fitted pseudo-first-order and pseudo-second-order models.

Heavy Metal Ion	Pseudo-First-Order			Pseudo-Second-Order		
	*q*_e1_ (mg g^−1^)	*k*_1_ (min^−1^)	*R* ^2^	*q*_e2_ (mg g^−1^)	*k*_2_ (g (mg min)^−1^)	*R* ^2^
Pb(II)	379.3	1.892	0.9995	381.4	0.0413	0.9998

### 2.4. Adsorption Isotherms

The isothermal adsorption experiments were carried out at different concentrations of Pb(II) in order to depict the interaction pathway between the PAA sponge and Pb(II). The removal efficiency of the PAA sponge varied with the initial concentration of Pb(II), as shown in Figure S9. With the increase in the concentration of Pb(II), the adsorption capacity of the PAA sponge increased gradually, while the removal efficiency decreased obviously. The decrease in the removal efficiency of Pb(II) was due to the limited adsorption sites as the number of Pb(II) increased. 

Furthermore, the Langmuir and Freundlich models were used to describe the adsorption and desorption isotherms of Pb(II). The Langmuir isotherm model assumes that the adsorption is monolayer and uniform, and also that there is no interaction between adsorbed molecules and the adsorption is dynamically at equilibrium [42]. The Freundlich model describes the adsorption equilibrium of a heterogeneous adsorbed surface [43].

(3) $$\frac{C_{e}}{q_{e}}=\frac{1}{K_{L}q_{m}}+\frac{C_{e}}{q_{m}}$$

(4) $$logq_{e}=logK_{F}C_{e}^{\frac{1}{n}}$$

where *q*_m_ (mg g^−1)^ and *q*_e_ (mg g^−1^) represent the maximum removal capacity and the removal capacity at equilibrium, respectively; *C*_e_ is the Pb(Ⅱ) concentration at equilibrium; *K*_L_ (L mg^−1^) is a Langmuir constant concerning adsorption energy; and n and *K*_F_ (mg g^−1^ (L mg^−1^)^1/n^) represent the Freundlich constant associated with adsorption intensity and adsorption capacity, respectively.

Figure 3C,D and Table 2 show the fitted curves and isothermal parameters fitted by Langmuir and Freundlich models. The correlation coefficients of the Langmuir isotherm adsorption model (*R*^2^ = 0.9972) are higher than those of the Freundlich model (*R*^2^ = 0.9304), implying that the adsorption process could be well described by the Langmuir model [44]. Though the equilibrium adsorption data of Pb(II) by PAA sponge at 25 °C can be fitted well with the Langmuir model, it is still hard to describe the adsorption process with a single adsorption model considering the structural features and the abundant functional groups of the PAA sponge [45,46]. The adsorption process might happen in a mixed manner, and we confirmed the conclusion in the following text. 

### 2.5. Recyclability of the PAA Sponge

**Figure 4:**
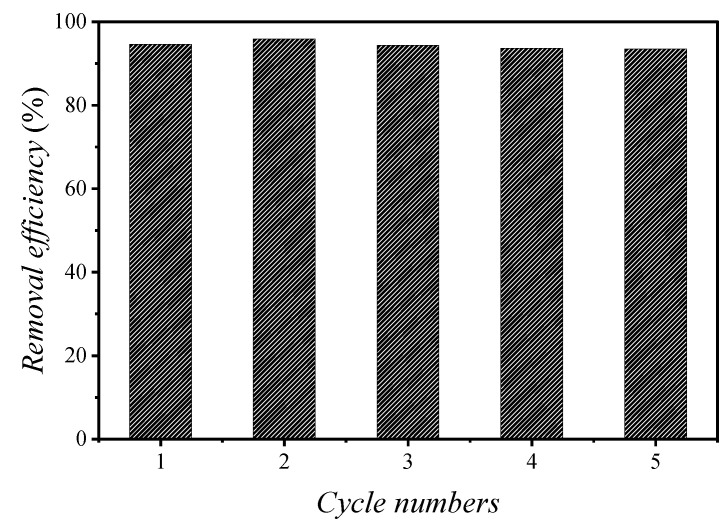
The recyclability of the PAA sponge after the adsorption of Pb(II).

Efficient desorption is very important for the recyclability of adsorbents. Therefore, we evaluated the recyclability of the PAA sponge under acidic conditions to release the adsorbed Pb(II). As illustrated in Figure 4, the adsorbed Pb(II) could be effectively released from the PAA sponge as treated by 0.1 M HCl aqueous solution. The PAA sponge showed excellent recyclability for multiple adsorption–desorption cycles, and the removal efficiency was still as high as 93% after five cycles.

### 2.6. Mechanism for the Adsorption of Pb(II) by PAA Sponge

**Figure 5:**
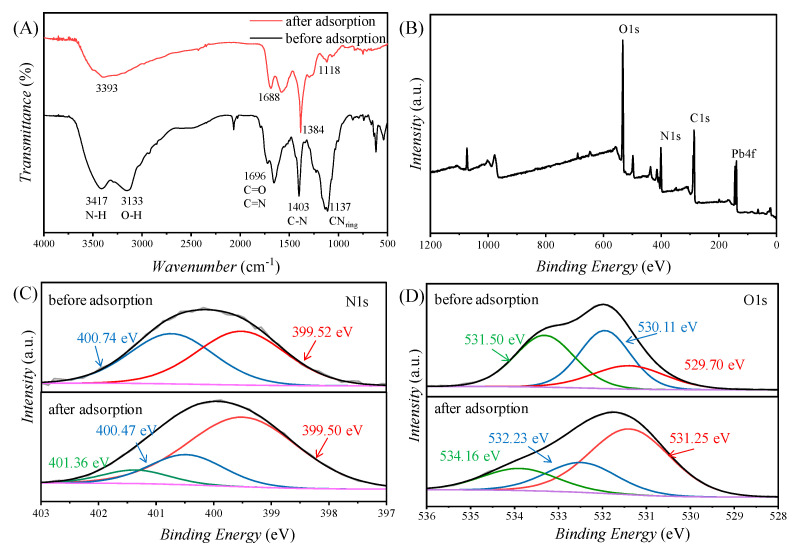
(**A**) FTIR spectra, (**B**) XPS survey and high resolution XPS spectra of (**C**) N1s and (**D**) O1s of PAA sponge after adsorption of Pb(II).

In order to understand the adsorption mechanism, FTIR spectra were obtained to analyze the changes in the characteristic absorption peaks of the functional groups before and after adsorption. As presented in Figure 5A, before the adsorption of Pb(II), the FTIR spectrum of the PAA sponge demonstrated that the adsorption peaks located at 1137, 1403, 1696, 3133 and 3417 cm^−1^ represented the bond stretching of CN_ring_, with stretching vibrations of C–N, C=O and C=N, O–H and N–H, respectively [47,48]. After the adsorption of Pb(II), the adsorption peak of C=N significantly weakened and shifted to 1118 cm^−1^, manifesting a strong interaction between the nitrogen species in the triazole ring and Pb(II), which could be further confirmed by the attenuation of the adsorption peak of N–H at 3417 cm^−1^ [49]. In addition, the disappearance of the adsorption peak of O–H demonstrated the electrostatic interaction between Pb(II) and carboxylic groups [50]. Overall, we can conclude that Pb(II) is adsorbed into the PAA sponge due to the strong interaction between the nitrogen species in the triazole ring and Pb(II), and electrostatic interaction between the carboxylic group and Pb(II). 

**Figure 6:**
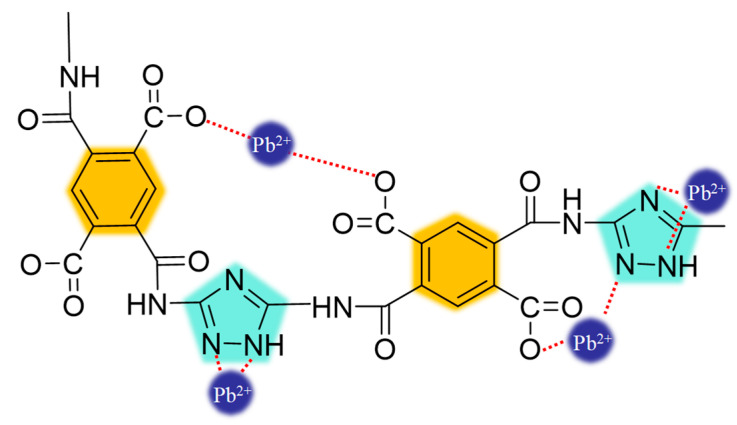
Schematic illustration of the adsorption mechanism of Pb(II) by PAA sponge.

To further confirm the adsorption mechanism, XPS analysis was conducted to investigate the interaction between Pb(II) and nitrogen and oxygen species in the PAA sponge. Figure 5B shows the XPS survey spectrum of Pb(II) adsorbed by the PAA sponge. Comparing with the XPS survey spectrum in Figure S10, the appearance of the adsorption peak of Pb4f located at 138.4 eV demonstrated that Pb(II) was effectively adsorbed by the PAA sponge. Subsequently, the high resolution spectra of N1s and O1s before and after the adsorption of Pb(II) were also analyzed, as illustrated in Figure 5C,D. The adsorption peak of N1s of the PAA sponge before the adsorption of Pb(II) could be divided into two peaks, indicating the existence of two chemical states of nitrogen (Figure 5C). Additionally, the peaks which appeared at 399.52 and 400.74 eV were attributed to the tertiary amine and secondary amine, respectively [51]. After adsorption, a new peak was observed at a binding energy of 401.36 eV, suggesting that the adsorption mechanism relied on the complexation between the nitrogen atom and Pb(II), where a pair of electrons in the nitrogen atom was supplied to the shared bond between nitrogen and Pb(II) [52]. In addition, the shift in the binding energy of nitrogen species further explained the change in the chemical environment caused by the sharing of electrons between the nitrogen atom and Pb(II) [53]. In addition, the high-resolution spectra of Pb4f and C1s were also presented in Figures S11 and S12. The O1s spectra of the PAA sponge before and after the adsorption of Pb(II) are shown in Figure 5D. The adsorption peak could be divided into three peaks, located at 531.39/531.25, 531.94/532.23 and 533.31/534.16 eV, which could be assigned to C=O, C–O and O–H bonds, respectively [54]. The shift and change in the intensity of the binding energy of the subdivided peaks proved that the coordination of Pb(II) with oxygen atoms occurred during adsorption [55]. Figure 6 vividly illustrates a possible adsorption mechanism between nitrogen atoms and the oxygen atoms of the PAA sponge forming strong coordination complexes with Pb(II). In summary, the efficient adsorption of Pb(II) by the PAA sponge was due to the synergy of physical adsorption and chemical adsorption. 

## 3. Materials and Methods

### 3.1. Materials

Pyromellitic dianhydride (PMDA, 99%) and 3,5-diamino-1,2,4-triazole (98%) were purchased from Aladdin Chemistry, Co., Ltd., Shanghai, China. DMSO-*d*_6_ was purchased from J&K Scientific Ltd., Shanghai, China. *N*, *N*-Dimethylformamide (DMF, AR), acetone (AR), Sodium hydroxide (NaOH, 99.99%), hydrochloric acid (HCl, 35%) and lead nitrate (Pb(NO_3_)_2_, 99%) were purchased from Sinopharm Chemical Reagent Co., Ltd., Shanghai, China. and used as received.

### 3.2. Experimental Section

#### 3.2.1. Synthesis of Poly(amic acid) (PAA)

To synthesize PAA, the 3,5-diamino-1,2,4-triazole (1.011 g, 10.00 mmol) dissolved in 20 mL of DMF was placed in an ice bath and the PMDA (2.204 g, 10.00 mmol) dispersion in 10 mL of DMF was added into the solution batch-by-batch in an hour. After stirring for another 2 h at ambient temperature, the polymerization was completed and the stock solution was obtained and stored at −20 °C. Subsequently, the stock solution was precipitated in acetone three times to afford PAA powder for further characterizations.

#### 3.2.2. Self-Assembly of the PAA into a Sponge

The PAA stock solution was diluted to 30 mg mL^−1^ by DMF. Deionized water (*v*_water_:*v*_DMF_ = 2:1) was added dropwise to the PAA solution at a speed of 5 mL h^−1^ by a peristaltic pump under stirring (250 rpm). DMF was removed by dialysis in deionized water. Then, the PAA sponge was obtained by freeze-drying under vacuum.

#### 3.2.3. Adsorption Experiments

Effect of pH values on the adsorption of Pb(II). In order to explore the influence of pH values on the adsorption of Pb(II), the pH values of Pb(II) aqueous solution (100 ppm, 25 mL) were adjusted with 0.1 M sodium hydroxide and hydrochloric acid. Subsequently, 5 mg of PAA sponge was added into the solution and stirred at 250 rpm for 24 h. All experiments were carried out at 25 °C, and 2 mL of the mixture was taken out via syringe and filtered with a 0.45 μm filter. After that, the filtrate was collected for inductively coupled plasma optical emission spectrometer (ICP-OES) analysis. The adsorption capacity of the PAA sponge for Pb(II) ions can be evaluated according to the following equations:

(5) $$q_{e}=\frac{(C_{0}-C_{t})V}{m}$$

(6) $$Removal\;efficiency=\frac{C_{0}-C_{t}}{C_{0}}\times 100\%$$

where *C*_0_ (mg L^−1^) and *C*_t_ (mg L^−1^) are the concentration of Pb(II) before and after adsorption; *m* (mg) is the mass of the PAA sponge; *V* (mL) is the volume of the Pb(II) solution; *q*_e_ is the adsorption capacity.

The determination of the removal efficiency and maximum adsorption capacity of Pb(II) at different dosages of the PAA sponge. PAA sponge in different dosages (2–8 mg) was added in 25 mL of 100 ppm Pb(II) solution at pH 5 and stirred at 250 rpm for 24 h. Then, 2 mL of the mixture was taken out via syringe and filtered with a 0.45 μm filter. The filtrate was collected for further analysis.

Adsorption kinetic studies. Briefly, 30 mL of 100 ppm Pb(II) solution was added to the 50 mL beaker, and 7.2 mg of PAA sponge was added into the solution. The mixture was stirred at 250 rpm for different times (2, 5, 10, 20, 30, 60, 120 min). Additionally, 2 mL of the mixture was taken out via syringe and filtered with a 0.45 μm filter. The filtrate was collected for ICP-OES measurement.

Adsorption isotherms. The adsorption isotherms were obtained by measuring the adsorption capacity of the PAA sponge at different Pb(II) concentrations (40, 60, 80, 100, 150, 200, 250 mg mL^−1^) at 25 ℃. The pH value and volume of the solution were adjusted to 5 and 25 mL, respectively. Then, 7.5 mg of PAA sponge was added into the solution and stirred for 24 h. The mixture was taken out via syringe and filtered with a 0.45 μm filter. The filtrate was collected for further analysis.

### 3.3. Characterizations

Fourier transform infrared spectroscopy (FTIR) analyses were carried out with a Germany TENSOR type II instrument, and proton nuclear magnetic resonance (^1^H NMR) spectra were recorded using a Bruker AVANCE III 400 MHz spectrometer with DMSO-*d*_6_ as a solvent. Nitrogen adsorption/desorption experiments were operated with Autosorb iQ to determine the specific surface area and pore distribution of the PAA sponge. The specific surface area of the PAA sponge was calculated with the Brunauer–Emmett–Teller (BET) method in the relative pressure (P/P_0_) range of 0.05 to 0.35, and the pore size distribution was calculated using the Barrett–Joyner–Halanda (BJH) model. Scanning electron microscope (SEM) images of the samples were observed by Hitachi Regulus 8100 at 5 kV and 10 μA. Transmission electron microscope (TEM) images were obtained by using Hitachi HT7700 with an acceleration voltage of 100 kV. X-ray photoelectron spectroscopy (XPS) spectra were received by Thermo SCIENTIFIC K-Alpha under vacuum. The concentrations of Pb(II) were determined by ICP-OES (PQ9000) and the instrument was calibrated using five standard solutions with concentrations of 0.25, 0.5, 1, 5 and 10 mg L^−1^.

## 4. Conclusions

In conclusion, a polymer self-assembly strategy was proposed to prepare a highly porous PAA sponge that showed abundant active functional groups for the efficient adsorption of Pb(II). The maximum adsorption capacity was 609.7 mg L^−1^, and the removal efficiency reached 97.2% with an adsorption capacity of 303.6 mg g^−1^. The adsorption kinetics study demonstrated the ultrafast adsorption of Pb(II) to reach a removal efficiency of 89.0% with an adsorption capacity of 370.7 mg g^−1^ within 2 min, and the adsorption process can be well fitted by both pseudo-first-order and pseudo-second-order kinetic models. In addition, the adsorption isotherm study revealed that the adsorption of Pb(II) follows the Langmuir isotherm model, indicating a monolayer adsorption manner. The PAA sponge also showed excellent recyclability, with a removal efficiency up to 93% after five cycles. Furthermore, FTIR and XPS analysis revealed that the adsorption mechanism is a combination of electrostatic interaction and the coordination and complexation between Pb(II) and nitrogen and oxygen atoms. In view of the high adsorption capacity and recovery of the PAA sponge, we believe that the PAA sponge developed in this study may be a promising candidate for the efficient removal of heavy metal ions.

## Figures and Tables

**Table 2:** Isothermal parameters and correlation coefficient fitted by Langmuir and Freundlich models.

Heavy Metal Ion		Langmuir Isotherm		Freundlich Isotherm		
	*K*_L_ (L mg^−1^)	*q*_m_ (mg g^−1^)	*R* ^2^	*K*_F_ (mg g^−1^ (L mg^−1^)^1/n^)	1/n	*R* ^2^
Pb(II)	0.1784	609.7	0.9972	118,700	0.2872	0.9304

## Data Availability

The data that support the findings of this study are available from the corresponding author upon reasonable request.

## Supplementary Materials

The following supporting information can be downloaded at: https://www.mdpi.com/article/10.3390/molecules28072897/s1, Scheme S1. Synthesis of PAA by condensation polymerization. Figure S1. FTIR spectrum of PAA. Figure S2. 1H NMR spectrum of PAA in DMSO-d6. Figure S3. TEM image of thin membranes formed by the self-assembly of PAA. Figure S4. Zeta potential of PAA sponge. Figure S5. (A) Nitrogen adsorption/desorption isotherm and (B) pore size distribution of PAA sponge. Figure S6. Effect of pH values on the zeta potentials of PAA sponge. Figure S7. (A) Top view and (B) side view of PAA sponge after adsorption of Pb(II). Figure S8. Effect of time on Pb(II) adsorption by PAA sponge. Figure S9. Effects of initial concentration on Pb(II) adsorption. Figure S10. XPS survey of PAA sponge. Figure S11. High-resolution Pb4f spectra of PAA sponge after adsorption of Pb(II). Figure S12. High-resolution C1s spectra of PAA sponge before and after adsorption of Pb(II).

## Sample Availability

Samples of the compounds are available from the authors.
