# Supplementary material for: Efficient Removal of Pb(Ⅱ) by Highly Porous Polymeric Sponges Self-Assembled from a Poly(Amic Acid)
Source: Molecules. 2023 Mar 23;28(7):2897. doi: 10.3390/molecules28072897 (PMC10095650; doi:10.3390/molecules28072897)
Supplement: Supplementary file 1 [file molecules-28-02897-s001.zip › molecules-2264125-supplementary.pdf]

Supplementary Information for

Efficient removal of Pb(II) by highly porous polymeric sponges self-assembled from a poly(amic acid)

Ying Leng [1], Kai Jin [1], Tian Wang [2], Xiaoyong Lai [1] and Hui Sun [1,*]

[1]State Key Laboratory of High-Efficiency Coal Utilization and Green Chemical Engineering, School of Chemistry and Chemical Engineering, Ningxia University, Yinchuan 750021, China

[2]Department of Chemistry, University of Washington, Seattle, WA 98195, USA

Scheme S1. Synthesis of PAA by condensation polymerization.

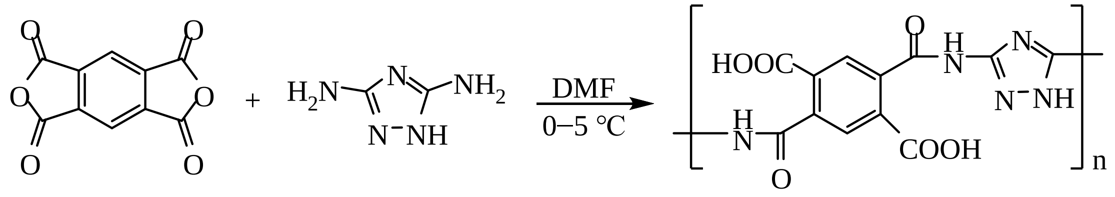

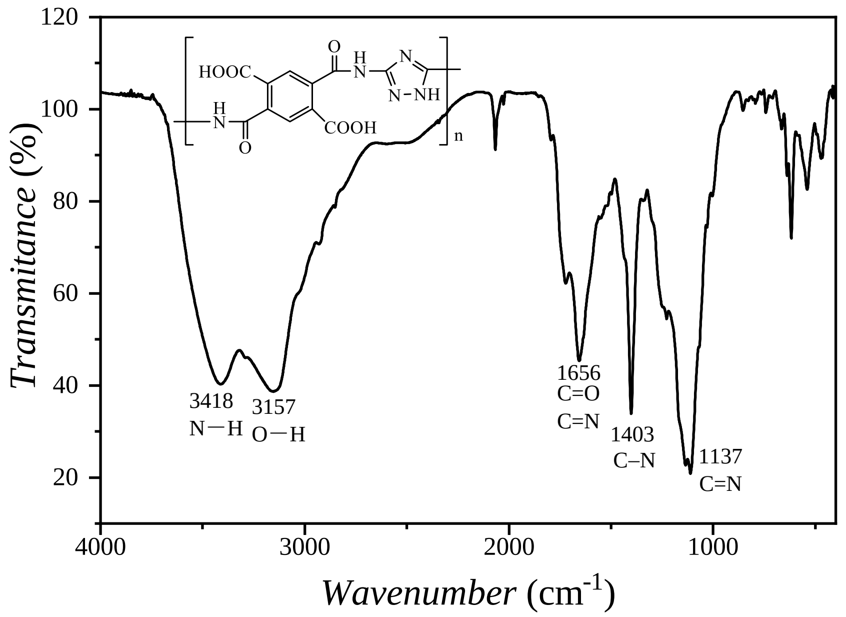

Figure S1. FTIR spectrum of PAA.

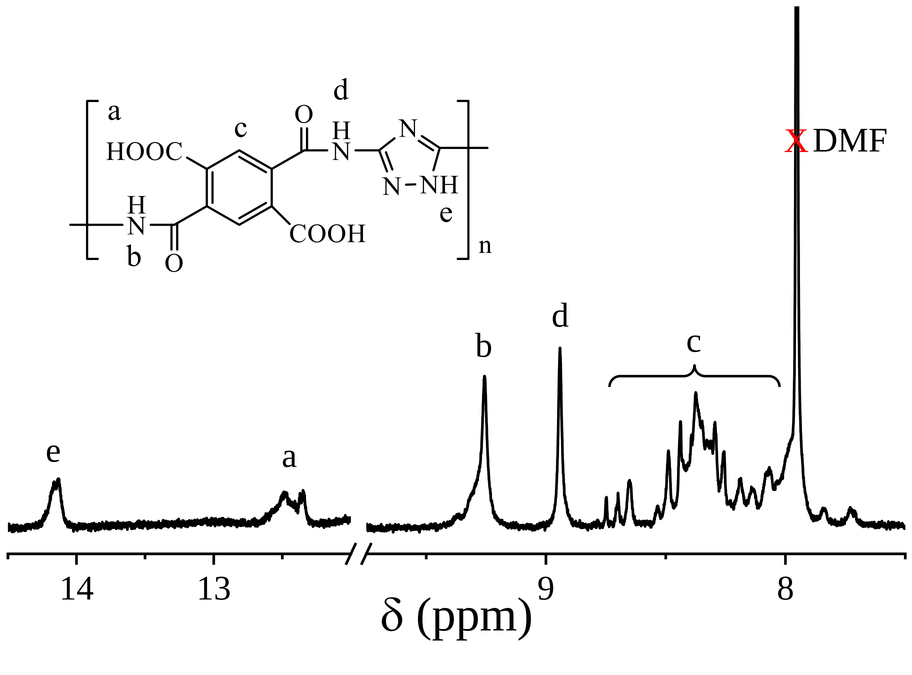

Figure S2. ^{1}H NMR spectrum of PAA in DMSO-d_6.

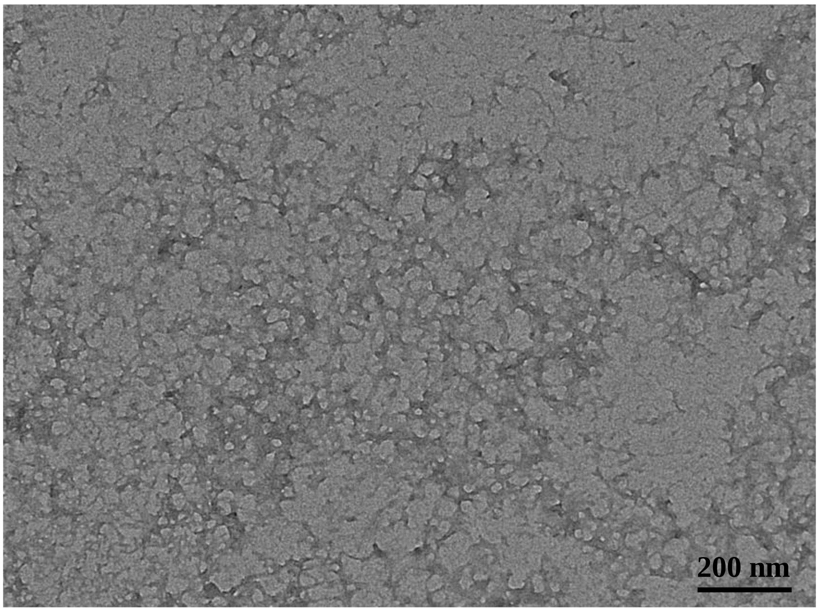

Figure S3. TEM image of thin membranes formed by the self-assembly of PAA.

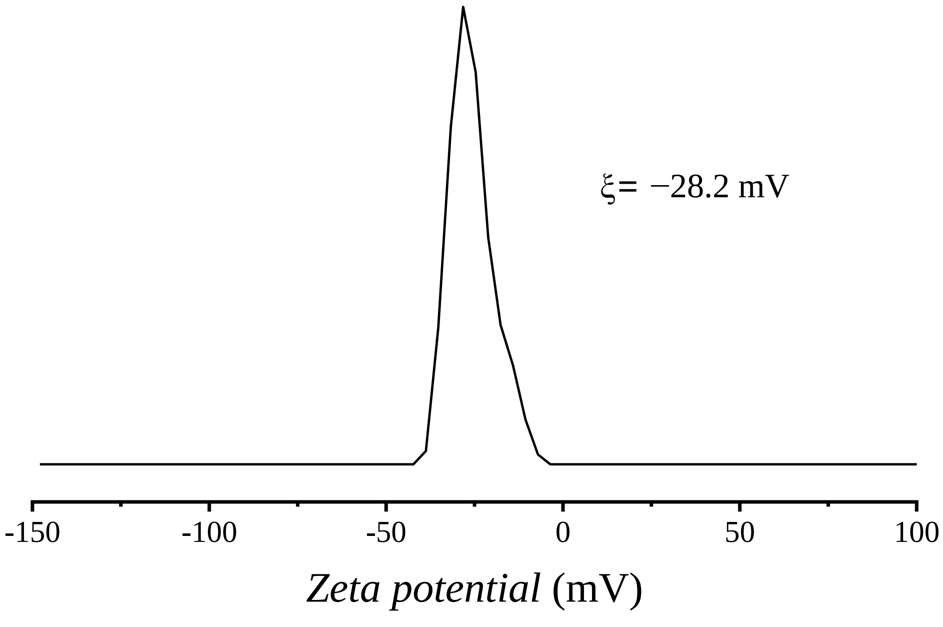

Figure S4. Zeta potential of PAA sponge.

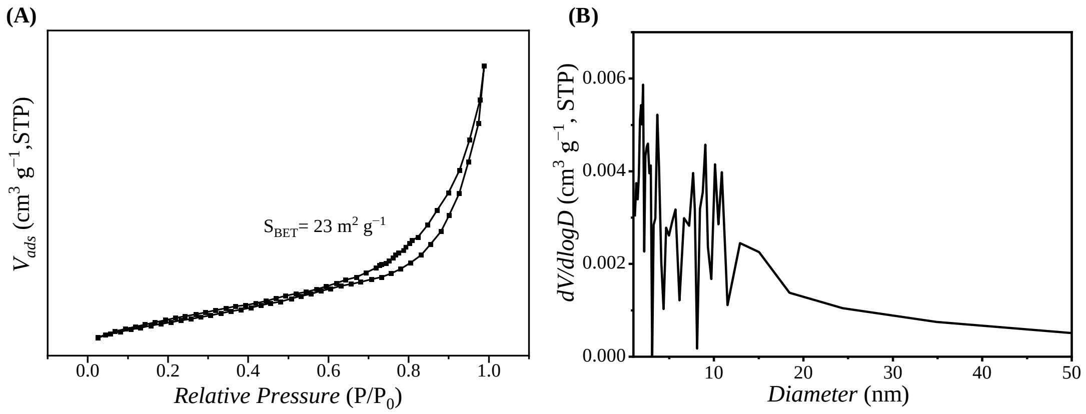

Figure S5. (A) Nitrogen adsorption/desorption isotherm and (B) pore size distribution of PAA sponge.

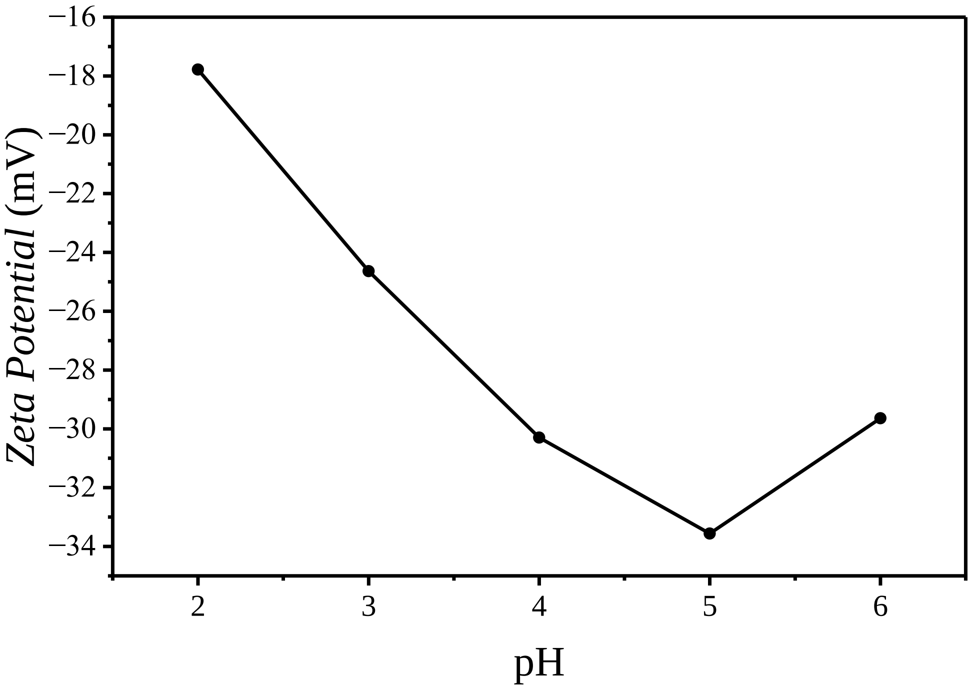

Figure S6. Effect of pH values on the zeta potentials of PAA sponge.

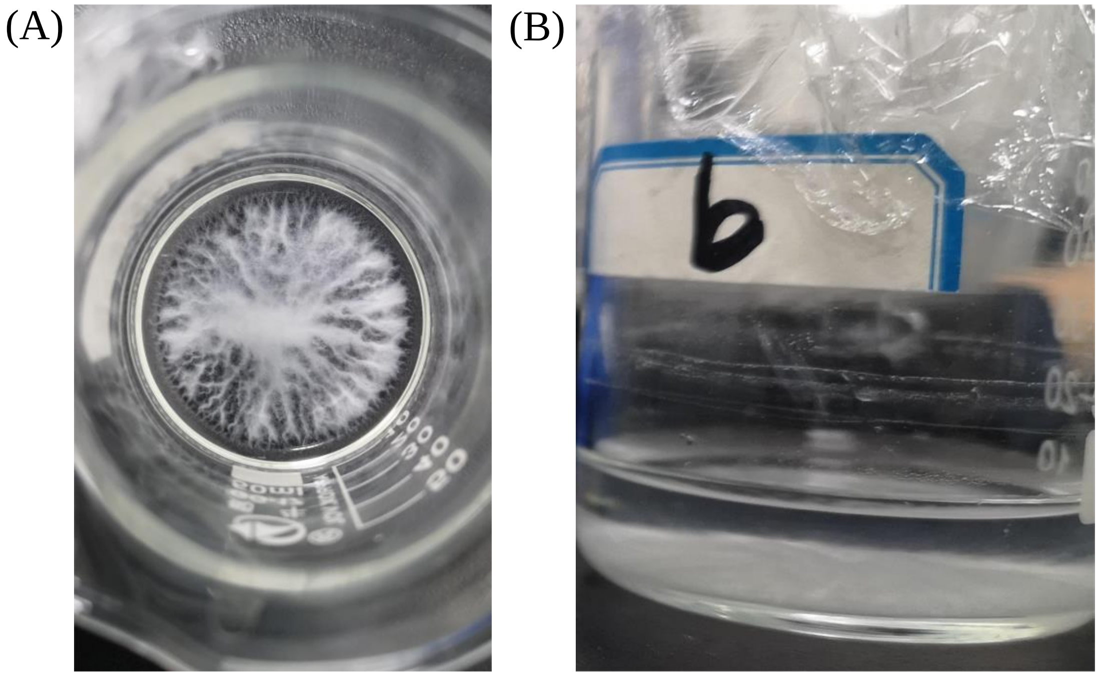

Figure S7. (A) Top view and (B) side view of PAA sponge after adsorption of Pb(II).

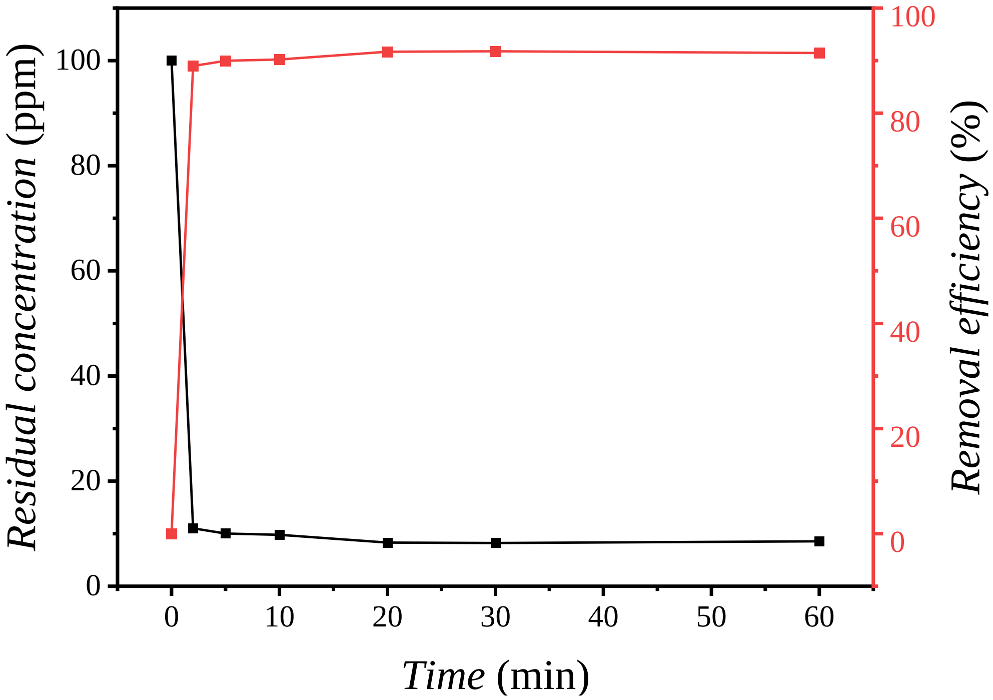

Figure S8. Effect of time on Pb(II) adsorption by PAA sponge.

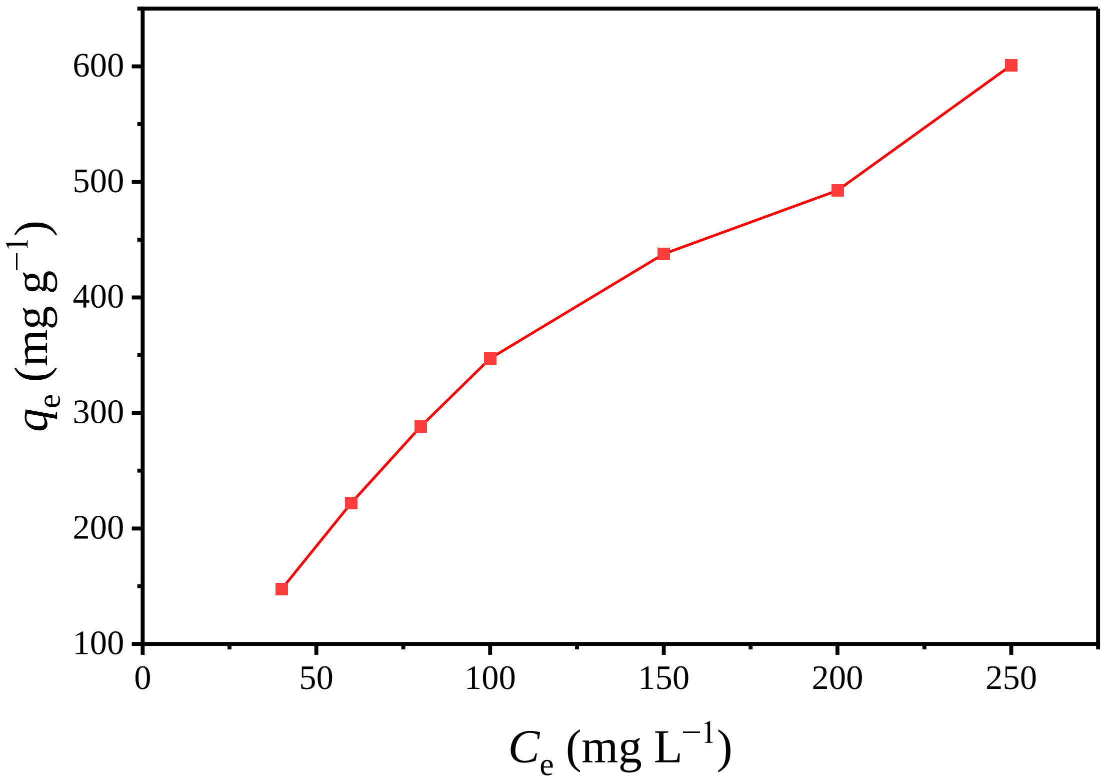

Figure S9. Effects of initial concentration on Pb(II) adsorption.

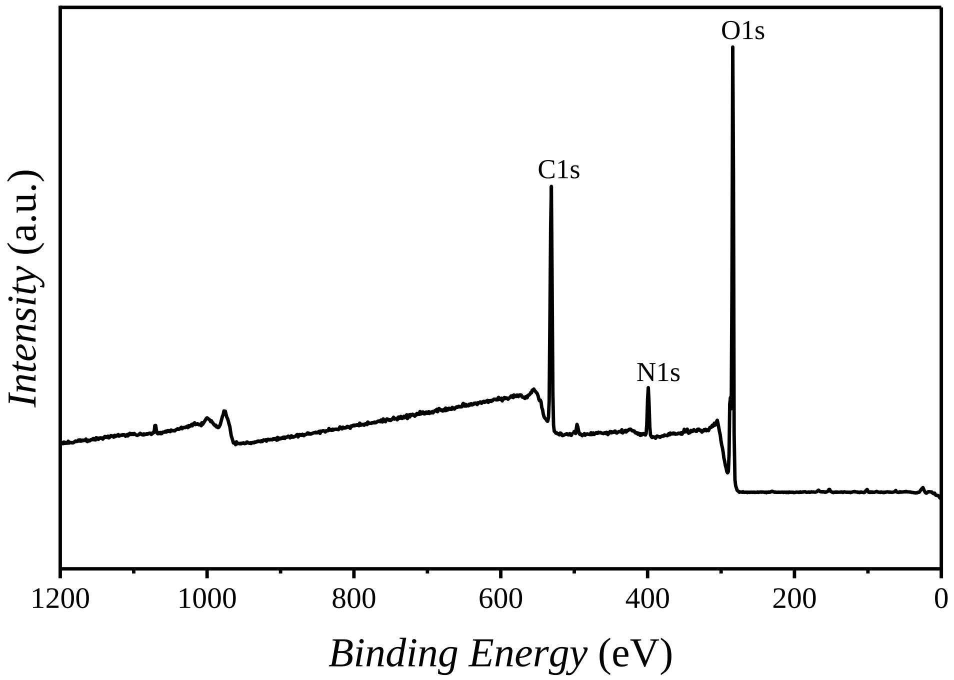

Figure S10. XPS survey of PAA sponge.

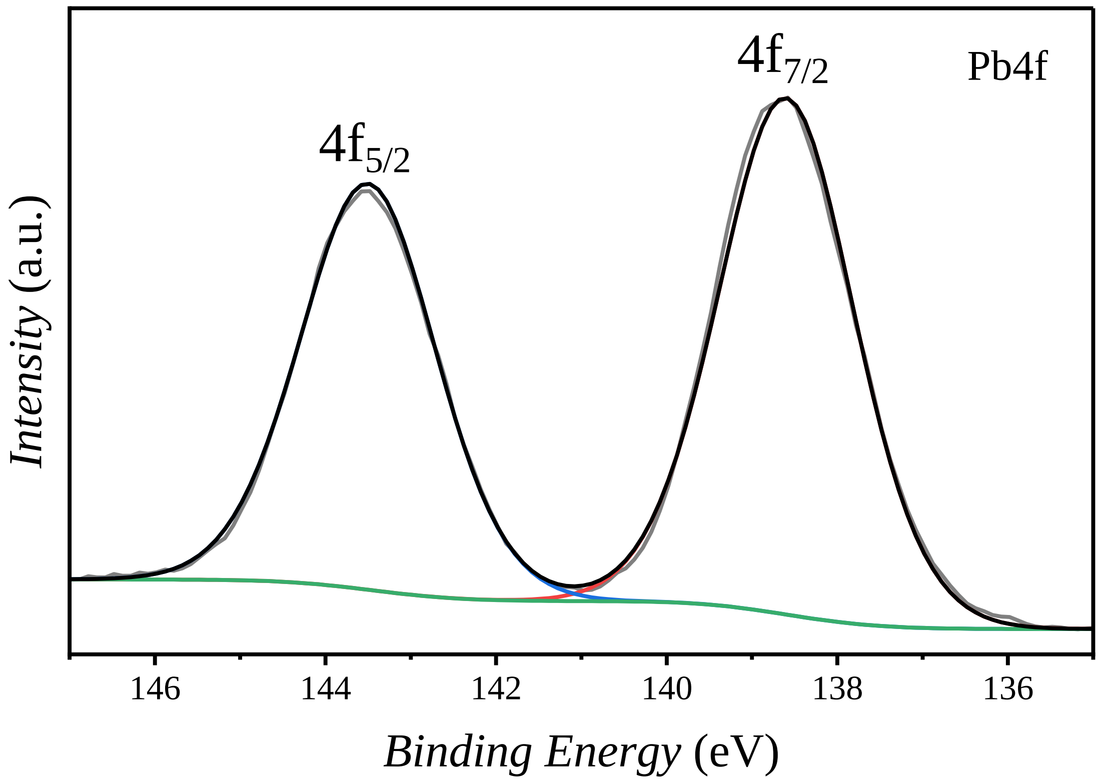

Figure S11. High-resolution Pb4f spectra of PAA sponge after adsorption of Pb(II).

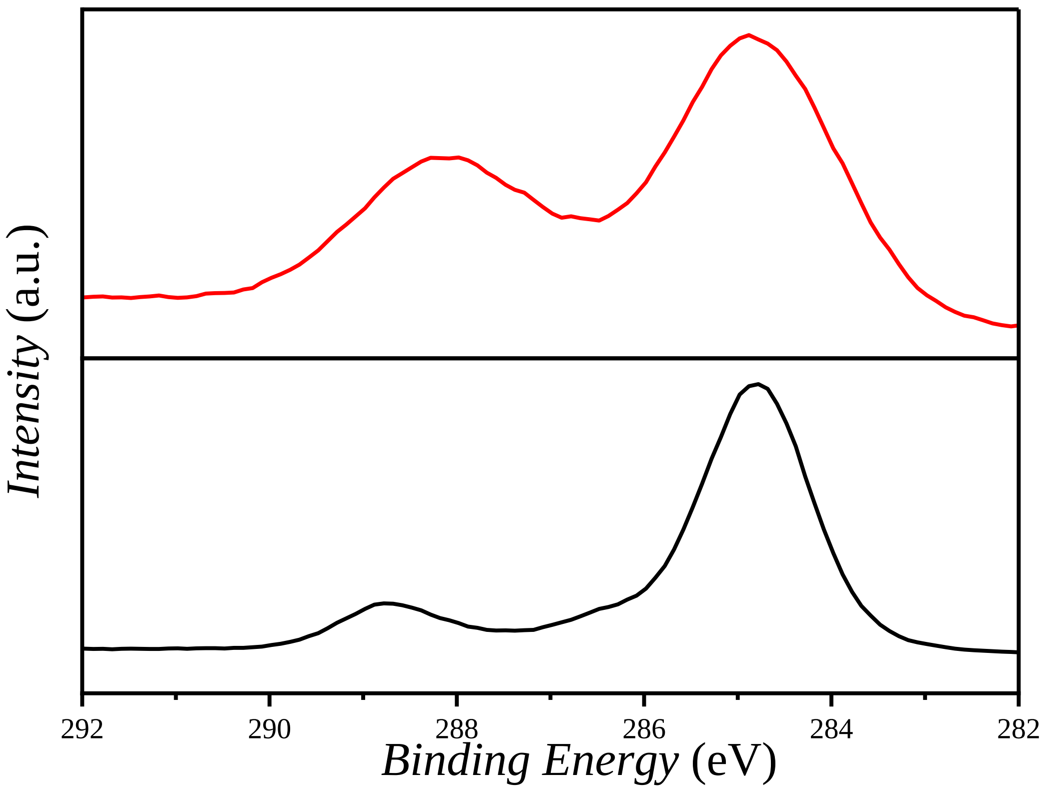

Figure S12. High-resolution C1s spectra of PAA sponge before and after adsorption of Pb(II).
